# Optimal process design space to ensure maximum viability and productivity in *Penicillium chrysogenum* pellets during fed-batch cultivations through morphological and physiological control

**DOI:** 10.1186/s12934-020-1288-5

**Published:** 2020-02-13

**Authors:** Lukas Veiter, Julian Kager, Christoph Herwig

**Affiliations:** 1 CD Laboratory on Mechanistic and Physiological Methods for Improved Bioprocesses, Vienna University of Technology, Gumpendorferstrasse 1a/166, 1060 Vienna, Austria; 2 Research Area Biochemical Engineering, Institute of Chemical, Environmental and Bioscience Engineering, Vienna University of Technology, Gumpendorferstrasse 1a/166, 1060 Vienna, Austria

**Keywords:** Filamentous fungi, *Penicillium chrysogenum*, Design of experiments, Flow cytometry, Viability, Morphology, Pellets, Multiple linear regression

## Abstract

**Background:**

Biomass growth of *Pencillium chrysogenum* is characterised by a distinct pellet morphology consisting of compact hyphal agglomerates. Fungal pellets are advantageous in industrial process control due to rheological advantages but lead to biomass degradation due to diffusional limitations of oxygen and substrate in the pellet’s core. Several fermentation parameters are known to affect key pellet characteristics regarding morphology, viability and productivity. Pellet morphology and size are affected by agitation. Biomass viability and productivity are tightly interlinked with substrate uptake and dissolved oxygen concentration.

**Results:**

The goal of this study was to study the impact of the fermentation parameters power input, dissolved oxygen content and specific substrate uptake rate on morphology, biomass viability and productivity. A design of experiments (DoE) approach was conducted and corresponding responses were analysed using novel morphological descriptors analysed by a previously established flow cytometry method. Results clearly display inverse correlations between power input and pellet size, specific morphological parameters related to pellet density can be increased in direct proportion to power input. Biomass viability and productivity are negatively affected by high specific substrate uptake rates.

**Conclusions:**

Based upon multiple linear regression, it was possible to obtain an optimal design space for enhanced viability and productivity at beneficial morphological conditions. We could maintain a high number of pellets with favourable morphology at a power input of 1500 W/m^3^. A sound compromise between viability and high productivity is possible at a specific glucose uptake rate of 0.043 g/g/h at dissolved oxygen levels of 40% minimum.

## Introduction

Cultivation strategies of filamentous fungi are characterized by specific fungal morphologies encompassing several forms ranging from homogeneously dispersed hyphae to dense agglomerates [22, 23]. Industrial bioprocesses using *Penicillium chrysogenum* favour the sphere-like pellet form where tightly packed mycelium forms a dense core surrounded by a looser ‘hairy’ region [9]. These spherical pellets lead to advantages for process control such as lower viscosity of the cultivation broth as it contains less tangled mycelia [23]. Lower mixing times and facilitated gas–liquid mass transfer enable higher cell densities during cultivation. However, pellet morphology also calls for a segregated view of biomass. Different pellet regions feature different characteristics: the outer pellet region shows higher metabolic activity than the pellet’s core which displays diffusional limitations mainly regarding oxygen [22]. For penicillin production, the pellet’s outer region is also the productive zone [12]. Consequently, the ideal pellet is characterised by (i) the largest possible viable outer zone and (ii) a rather loose morphology with a large ‘hairy’ region [9], at the same time (iii) being dense and compact enough to ensure all the rheological advantages of pellet morphology.

From the perspective of morphology, effects of agitation have been extensively described [21–23]. Generally, pellet size as well as pellet quantity can be lowered by increased agitation [9, 19] as well as morphological aspects such as compactness [2]. Apart from influences on morphology, higher agitation also increases the power input into the system and by extend affects mixing time and k_L_a [5]. It should be noted that the factor power input by itself only depicts average agitation conditions inside a bioreactor. In the case of filamentous fungi, stirrer type and geometry are also highly relevant due to drastic differences in shear forces and uniform energy dissipation [22]. To avoid destructive forces on pellets, low-shear impellers like the pitched-blade type are preferable to conventional Rushton turbines if possible [2].

The characteristics of diffusional limitations of oxygen and nutrients within fungal pellets are essential when dealing with pellet morphology. Hille et al. [6] reported sharp decreasing oxygen concentration profiles along the pellet radius. Mass transport in pellets is commonly described by the effective diffusion coefficient $D_{eff}$ according to Eq. (1) with diffusion factor $f_{D}$ and molecular diffusion coefficient $D_{mol}$. $D_{eff}$ is dependent on porosity $\varepsilon_{P}$ whereas $\varepsilon_{P}$ or $f_{D}$ is changing along the pellet radius in the case of an inhomogeneous porosity [7].

1 $$D_{eff} = f_{D} *D_{mol} = \varepsilon_{P} *D_{mol}$$

$D_{eff}$: effective diffusion coefficient [m^2^ s^−1^], $D_{mol}$: molecular diffusion coefficient [m^2^ s^−1^], $f_{D}$: diffusion factor [−], $\varepsilon_{P}$ porosity [−].

These pellet characteristics can be defined by the terms porosity $\varepsilon_{P}$ or ‘pellet compactness’ [20], a more ‘compact’ pellet is fundamentally dense and features a smaller ‘hairy’ region. Studies in diffusivities and mass fluxes employing microelectrodes and evaluation of oxygen profiles indicate a negative correlation between compactness and $D_{eff}$ [7]. Consequently, a ‘compact’ pellet will lead to diffusional limitations which in turn will lead to a deterioration of viability. However, Hille et al. [7] also mention that while penetration of oxygen is facilitated in less compact pellets, also the amount of biomass supplied with oxygen is lower. Therefore, at-line monitoring of pellet viability in addition to pellet compactness is necessary for robust process control.

Additionally, there are interlinks with substrate consumption: substrate oxidation inside the pellet causes rapid consumption of the diffused oxygen which makes substrate availability a critical process parameter regarding oxygen limitation. During limiting substrate regimes oxygen penetration depth can be influenced based on different specific substrate uptake rates [1]. Being the main trigger for productivity [3], substrate limiting regimes are widely used in state-of-the-art production processes [1]. Several articles describe the relation of specific growth rate, substrate availability and productivity [3, 14, 18]. However, knowledge on the effect of oxygen penetration as a function of substrate availability is still scarce. By studying these influences, the interlinks with pellet viability can be further addressed.

In this publication, we used a design of experiments (DOE) approach to analyse factors affecting pellet morphology and viability in *P. chrysogenum* fed-batch processes using novel morphological descriptors. Subsequently we performed optimization of said factors employing multiple linear regression to achieve enhanced biomass viability and productivity. As potentially influencing factors we selected the power input (P/V), dissolved oxygen content (dO_2_) and specific substrate uptake rate (q_s_). Morphological and physiological responses were analyzed by a previously established flow cytometry method. These responses depict pellet size and two novel morphological descriptors: pellet compactness (C) and viable pellet layer (vl). Statistical evaluation of fermentation results provided insights into the influence of examined factors on the measured responses. Combining the obtained information, optimal operating ranges for optimised pellet characteristics and productivity will be presented to define a design space ensuring an efficient and productive fed-batch process.

## Materials and methods

### Strain

Spore suspensions of the P-14 *P. chrysogenum* candidate strain for penicillin production descending from the P-2 *P. chrysogenum* candidate strain (American Type Culture Collection with the access number ATCC 48271) were kindly provided by Sandoz GmbH (Kundl, Austria) and used for all experiments.

### Bioreactor cultivations

All cultivations were performed in a DASGIP Mini parallel reactor system (working volume 4*2.0 L, Eppendorf, Germany). The batch was inoculated with approximately 2∙10^8^ spores/L. During batch phase pH was not controlled. The end of the batch was defined per default as an increase in pH of 0.5 by convention. After the batch, the broth was diluted with fed-batch medium (15% broth, 85% medium) and fed-batches were started. Details on batch and fed-batch media can be found in Posch and Herwig [15].

The fed-batch process lasted for approximately 150–170 h. Temperature was maintained at 25 °C and pH was kept constant at 6.5 ± 0.1 by addition of 20% (w/v) KOH or 15% (v/v) H_2_SO_4_, respectively. pH was measured using a pH probe (Hamilton, Bonaduz, Switzerland). After additional 12 h nitrogen and phenoxyacetate feeds were started at constant rates (6.5 ml/h for nitrogen and 2 ml/h for phenoxyacetate).

A feed-forward controller was implemented to maintain a constant biomass specific glucose uptake rate (q_s_). The glucose feed was adjusted based on Eq. (2) which includes the actual biomass concentration within the bioreactor estimated by real-time model simulation of a literature model of *P. chrysogenum* [10, 11]. The original model was modified by only using the description of growing tips (c_A0_) and non-growing regions (c_A1_) and adding phenoxyacetic acid (c_POX_). The resulting state vector × contained V(t), c_A0_ (t), c_A1_ (t), the glucose c_S_ (t) concentration, the penicillin concentration (c_Pen_ (t)) and c_POX_ (t). In sum, the model contained 19 parameters, which were determined by log-likelihood maximization between historic experiments and model simulations. Based on model simulations and measured oxygen uptake and carbon evolution rate a particle filter was used to estimate the overall biomass concentration according to Eq. (3) comprising growing and non-growing biomass regions. Exact model equations, parameter values and further details on the state estimation algorithm can be found in Stelzer et al. [17] and Kager et al. [8].

2 $${\text{F}}_{{\left( {\text{t}} \right)}} = \frac{{{\text{qs}}_{{\left( {\text{t}} \right)}} * {\text{X}}_{{\left( {\text{t}} \right)}} * {\text{V}}_{{\left( {\text{t}} \right)}} }}{{{\text{C}}_{\text{S}} }}\,\,\left[ {\text{L/h}} \right]$$

3 $${\text{x}}\left( {\text{t}} \right) = {\text{c}}_{{{\text{A}}0}} + {\text{c}}_{{{\text{A}}1}}\, \left[ {\text{g/L}} \right]$$

F(t): feed flow rate [L/h] at time (t), q_s_(t): biomass specific substrate uptake rate [g/g] at time point (t), x(t): Biomass concentration [g/L] at time (t), V(t): reactor volume [L] at time (t), c_s:_ substrate concentration in feed [g/L], c_A0_ (t): concentration of growing tips [g/L] at time (t), c_A1_ (t): concentration of non-growing tips [g/L] at time (t), c_Pen_ (t): penicillin concentration [g/L] at time (t), c_POX_ (t): phenoxyacetic acid concentration [g/L] at time (t).

The stirrer was equipped with three six bladed Rushton turbine impellers, of which two were submersed and one was installed above the maximum liquid level for foam destruction. Aeration was controlled at 1 vvm in batch and initial fed-batch with mass flow controllers (Vögtlin, Aesch, Switzerland). Dissolved oxygen concentration was measured using a dissolved oxygen probe (Hamilton, Bonaduz, Switzerland) and controlled between 40% and 90% during batch and at the set-points 5.0, 22.5% or 40.0% during fed-batch, via adjustment of the gas mix using pressurized air, nitrogen and oxygen. The agitation conditions were maintained at 325–500 rpm stirring speed in the batch phase. For the duration of the entire fed-batch phase power input (P/V) was calculated according to equations by Rutherford et al. [16], specifically Eqs. (4 and 5), and controlled at the set-points 370, 1535 or 2000 W/m^3^ via adjustment of stirrer speed.

4 $${\text{P}}/{\text{V}} = \rho * {\text{N}}_{\text{P}} * {\text{n}}^{3} * {\text{d}}_{{}}^{5}$$

5 $${\text{N}}_{\text{P}} = 6.57 - 64.771 *\left( {\frac{{{\text{b}}_{\text{t}} }}{\text{d}}} \right)$$

$\rho$: density medium [1022 kg/m^3^], $N_{P}$: Newton number [−], $n$: agitation speed [rpm], $d$: impeller diameter [45 mm], $b_{t}$: blade thickness [1.25 mm].

CO_2_ and O_2_ concentration in the off gas were analysed with an off-gas analyser (DASGIP MP8, Eppendorf AG, Germany), using infrared and paramagnetic principle respectively (Bluesens GmbH, Germany), which were used as inputs for the biomass state observer as described in Stelzer et al. [17], Kager et al. [8].

### Experimental design of bioreactor cultivations

**Fig. 1:**
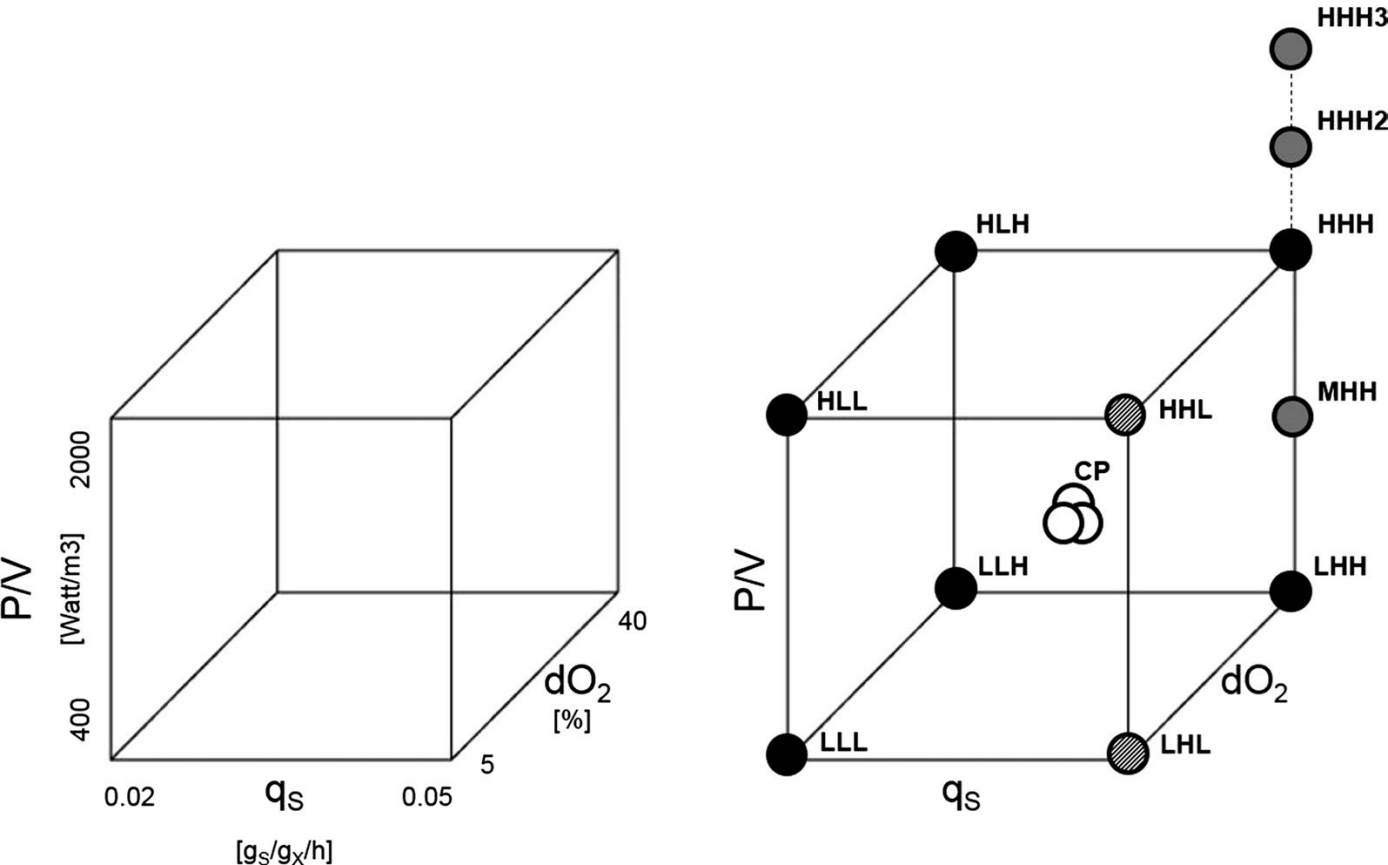
Experimental design of bioreactor cultivations (left). Factor ranges of bioreactor cultivations including nomenclature (right): additional cultivations exceeding the standard number of experiments along the P/V range (grey circles), centre-points (white circles), cultivations were setpoints could not be sustained due to external constraints (painted circles)

A full factorial design including power input (P/V), dissolved oxygen (dO_2_) concentration and availability of limiting substrate in the form of specific substrate uptake rate (q_s_) was employed. The design for all bioreactor cultivations is depicted in Fig. 1, in total 11 + 3 cultivations were performed. All relevant factors and respective nomenclature are summarized in Table 1. Multiple linear regression analysis was performed using the software MODDE10 (Umetrics, Umeå, Sweden).

**Table 1:** Multivariate experimental design of 11 + 3 bioreactor cultivations, nomenclature and factors with mean values over process time including standard deviation

Name	P/V [W/m^3^]	Q_s_ [g_s_/g_x_/h]	dO_2_ [%]
LLL	0370 ± 10	0.017 ± 0.003	5.0 ± 1.5
HLL	2012 ± 20	0.018 ± 0.001	5.0 ± 1.6
LHL	0370 ± 12	0.040 ± 0.012	5.0 ± 3.1
HHH1	1900 ± 15	0.042 ± 0.004	40.0 ± 6.5
LLH	0370 ± 11	0.015 ± 0.004	40.0 ± 5.2
MMH	1535 ± 20	0.038 ± 0.003	40.0 ± 4.1
LHH	0370 ± 14	0.045 ± 0.004	40.0 ± 5.2
HHH2	2700 ± 15	0.049 ± 0.005	40.0 ± 7.5
CP1	1535 ± 11	0.034 ± 0.005	22.5 ± 6.6
CP2	1535 ± 11	0.033 ± 0.005	22.5 ± 5.0
CP3	1535 ± 12	0.035 ± 0.003	22.5 ± 6.9
MHH	1113 ± 14	0.054 ± 0.005	40.0 ± 5.4
CP4	1535 ± 32	0.026 ± 0.003	22.5 ± 3.9
HHH3	3000 ± 34	0.050 ± 0.005	40.0 ± 9.5

The center point represents standard operation conditions (P/V = 1500 W/m^3^, dO_2_ = 22.5%, q_s_ = 0.035 g/g/h). In order to generate a sufficient morphological response in the pellet fraction we used a wide range of P/V set-points based on preliminary experiments with 1500 W/m^3^ as standard set-point. In two cultivations we exceeded the experimental boundary of 2000 W/m^3^ to generate further morphological effects. To maintain the P/V set-points the dO_2_ was solely controlled via the in-flow gas mix composition. In addition, we employed various q_s_ and dO_2_ set-points to test our hypothesis: the specific substrate uptake rate affects the viable pellet layer due to inter-dependency of oxygen and substrate consumption. Note that the highest q_s_ could not be sustained at low dO_2_ for an entire cultivation (LHL and LLH as displayed in Fig. 1).

### Flow cytometry

Samples from fed-batch cultivations were diluted 1:10 into phosphate buffered saline (50 g/L of 2.65 g/l CaCl_2_ solution, 0.2 g/L KCl, 0.2 g/L KH_2_PO_4_, 0.1 g/L MgCl ∙ 6 H_2_O, 8 g/L NaCl and 0.764 g/L Na_2_HPO_4_ + 2 H_2_O) and stained with propidium iodide (Sigma Aldrich, St. Louis, Missouri/USA; 20 mM stock dissolved in DMSO ≥ 99.9%, diluted with phosphate buffered saline to a final concentration of 20 µM) and fluorescein diacetate (Sigma Aldrich, St. Louis, Missouri, USA; stock solution of 5 g/L dissolved in acetone ≥ 99.9% to a final concentration of 5 mg/L). After incubation of 5 min, the sample was further diluted (1:100 in the same buffer) for flow cytometric analysis. Metabolic activity is shown by FDA treatment resulting in green fluorescence through esterase activity. PI fluorescence is a result from DNA intercalation in cells with compromised membranes [21].

A CytoSense flow cytometer (CytoBuoy, Woerden, Netherlands) with two forward scatter (FSC), one sideward scatter (SSC) and two fluorescence channels (green, red) was used for particle analysis. The implemented laser had a wavelength of 488 nm. The configuration of the filter set was 515–562 ± 5 nm for the green fluorescence channel (FL-green, used for fluorescein diacetate) and 605–720 ± 5 nm for the red fluorescence channel (FL-red, used for propidium iodide). The device was equipped with a PixeLINK PL-B741 1.3MP monochrome camera for in flow image acquisition. For data treatment, the software CytoClus3 (CytoBuoy, Woerden, Netherlands) and a custom-programmed Matlab 2016b script (MathWorks, Nattick, Massachusetts, USA) were used.

**Fig. 2:**
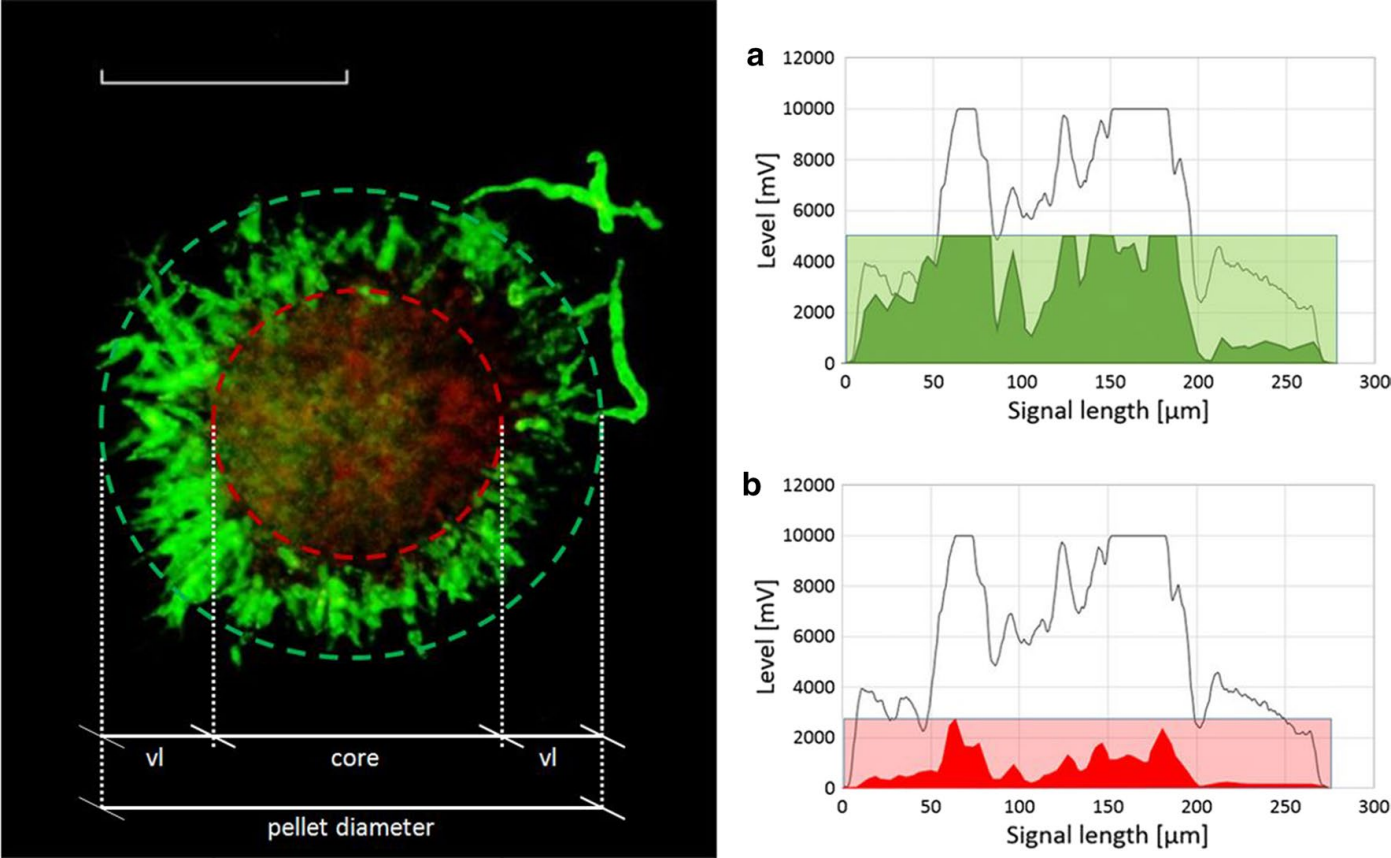
Left: confocal microscopy of pellet with enhanced contrast depicting pellet diameter, viable layer (vl), compact core region (red circle) and hairy outer region (green circle). White line = 50 µm. Right: corresponding signal profiles from flow cytometry depicting **a** viable area across pellet diameter and **b** degraded area in the pellet’s core according to Veiter and Herwig [20]

The flow cytometry method allows for determination of the following responses as depicted in Fig. 2: volume ratio of pellets in relation to all morphological classes (= pellet ratio in  %), average size of pellets (pellet size in µm), pellet compactness (no unit) and viable pellet layer (vl in µm). Further details on the method including data evaluation can be found in Veiter and Herwig [20].

**Fig. 3:**
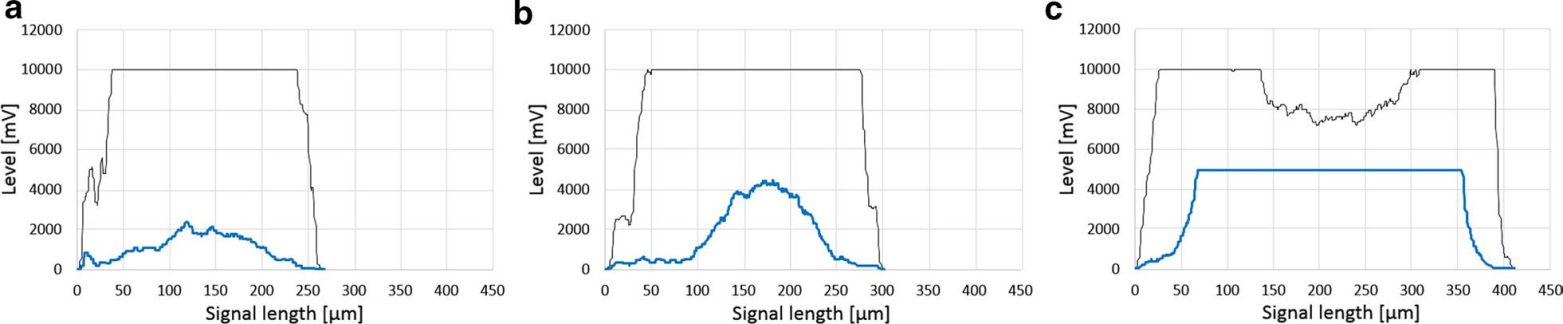
Spatially resolved pellet signal profiles, FSC signal (black) and SSC signal (blue). Pellet with low compactness (**a**) according to SSC signal. Pellet with high compactness according to SSC signal (**b**). Saturated SSC signal and pellet breakage according to FSC signals at elevated pellet diameters and high overall compactness (**c**)

As depicted in Fig. 3, compactness can be obtained from the analysis of SSC signal length in combination with particle size, hereafter termed “Compactness according to SSC” and calculated according to the following equation:

6 $${\text{Compactness }}_{\text{SSC}} { = }\frac{{{\text{Length of SSC signal}}\, \left[ {\upmu {\text{m}}} \right]}}{{{\text{Particle diameter }}\,\,\left[ {\upmu {\text{m}}} \right]}}$$

To further estimate pellet viability and demonstrate the relation of viable layer to pellet size, a viability factor was calculated according to Eq. (7).

7 $${\text{Viability factor vf}}\, \left[ - \right] = \frac{{2 * {\text{viable layer}}\,\,\left[ {\upmu {\text{m}}} \right]}}{{{\text{pellet size }}\,\,\left[ {\upmu {\text{m}}} \right]}}$$

### HPLC analytics

High performance liquid chromatography (HPLC) using a Thermo Scientific UltiMate 3000 system (Thermo Fisher Scientific, Massachusetts, United States) with a Zorbax Eclipse AAA C18 column (Agilent Technologies, Santa Clara, USA) was used to quantify penicillin V and phenoxyacetic acid concentration with a buffer as described elsewhere (Ehgartner, Fricke [19]). A flow rate of 1.0 ml/min was applied and the temperature of the column oven was 30 °C. The UV/VIS detector for determining penicillin and phenoxyacetic acid peaks via absorption was set to 210 nm.

## Results and discussion

In the following, results from multiple linear regression will be presented as a preliminary overview. A detailed discussion on the effects of factors power input (P/V), specific substrate uptake rate (q_s_) and dissolved oxygen content (dO_2_) on morphology, viability and productivity is available in the subsequent sections “Impact of power input on morphology”, “Impact of factors on viability” and “Interlink between productivity and specific substrate uptake”. These findings provide the base for an optimal process design which is summarized in section “Optimal process design space”.

### Multiple linear regression

**Fig. 4:**
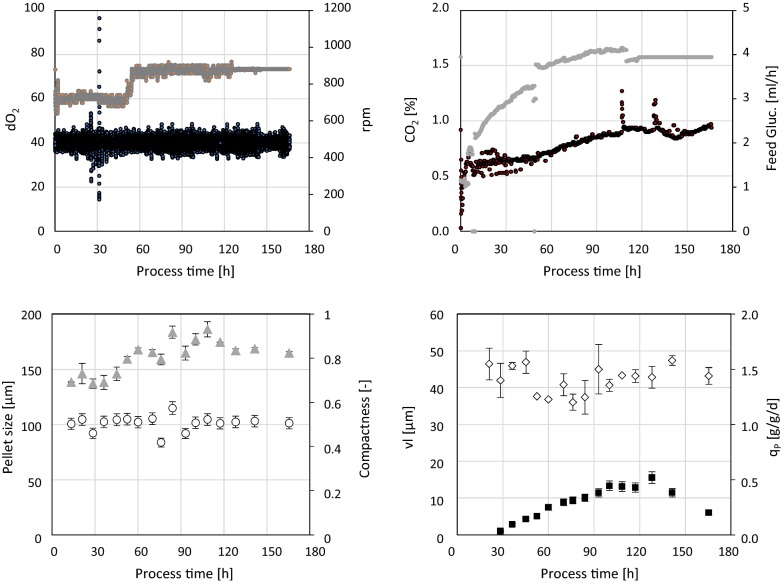
Top: process data across process time: dO_2_ (black), agitation via rpm (grey), CO_2_ in off-gas (black), glucose feeding rate (grey). Bottom: responses across process time: pellet size (grey triangles), compactness (circles), viable layer (circles) and specific productivity (black rectangles)

The effects of process parameters on DoE responses across process time (see Table 1) are exemplarily displayed for cultivation LLH in Fig. 4: due to low a P/V distinct effects on pellet size and pellet compactness are visible. Furthermore, low q_s_ and simultaneously high dO_2_ affect viability and productivity alike. All these interactions were analysed will be discussed in detail in the following.

All responses were subjected to single factor ANOVA analysis (α = 0.05) to test for statistically significant results rather than noise indicated by p-values of less than 0.05. For all responses the F-value is greater than the F-critical value for the alpha level selected (0.05), indicating significantly different means in the samples which thus belong to an entirely different population. Detailed information on the results from ANOVA analysis can be found in Additional file 1: Table S1.

To subsequently analyse all morphological and physiological responses considered in this study in a combined fashion, multiple linear regression (MLR) was used to study the effects on responses: mean pellet size, mean pellet compactness (C), mean viable pellet layer (vl) and mean specific productivity (q_P_). Mean values of each response over the entire process time were considered for this. Table 2 summarizes factors and responses generated from all 14 bioreactor cultivations performed in this study as depicted in Fig. 1. Model statistics are summarised and specified as a summary-of-fit displayed in Table 3. Overviews detailing summary-of-fit for all responses are displayed in Additional file 1: Figs. S1–S4.

**Table 2:** Design space, factors and responses including standard deviations from full factorial study comprising 11 + 3 additional bioreactor cultivations

Name	P/V [W/m^3^]	Q_s_ [g_s_/g_x_/h]	dO_2_ [%]	Mean pellet size [µm]	Mean C [−]	Mean vl [µm]	Mean q_p_ [g_P_/g_X_/d]
LLL	370	0.017	5.0	150.7 ± 9.5	0.51 ± 0.02	31.5 ± 5.1	0.18 ± 0.12
HLL	2012	0.018	5.0	129.3 ± 4.5	0.88 ± 0.03	38.6 ± 3.0	0.19 ± 0.09
LHL	370	0.040	5.0	183.1 ± 9.3	0.52 ± 0.02	22.1 ± 9.8	0.02 ± 0.01
HHH1	1900	0.042	40.0	135.7 ± 9.0	0.61 ± 0.03	33.6 ± 3.1	0.41 ± 0.24
LLH	370	0.015	40.0	161.7 ± 9.0	0.48 ± 0.02	40.1 ± 4.2	0.29 ± 0.23
MMH	1535	0.038	40.0	136.5 ± 5.8	0.48 ± 0.01	33.1 ± 3.0	0.38 ± 0.17
LHH	370	0.045	40.0	142.3 ± 5.3	0.44 ± 0.03	28.9 ± 2.9	0.29 ± 0.17
HHH2	2700	0.049	40.0	128.7 ± 5.1	0.85 ± 0.02	31.7 ± 2.3	0.34 ± 0.21
CP1	1535	0.034	22.5	136.5 ± 8.0	0.49 ± 0.05	36.4 ± 3.1	0.44 ± 0.19
CP2	1535	0.033	22.5	136.1 ± 5.8	0.48 ± 0.02	33.3 ± 3.5	0.40 ± 0.16
CP3	1535	0.035	22.5	129.6 ± 4.5	0.49 ± 0.03	38.1 ± 3.0	0.48 ± 0.21
MHH	1113	0.054	40.0	128.0 ± 5.1	0.49 ± 0.02	28.0 ± 3.1	0.13 ± 0.09
MMM	1535	0.026	22.5	141.7 ± 6.9	0.50 ± 0.03	40.1 ± 4.2	0.29 ± 0.13
HHH3	3000	0.050	40.0	120.8 ± 5.1	0.88 ± 0.03	31.0 ± 3.2	0.30 ± 0.19

**Table 3:** Summary of fit for model responses

Model response	Model parameter (95% significance level)	R2	Q2	Model validity	Reproducibility
Pellet size	P/V	0.60	0.43	0.50	0.93
Pellet compactness	P/V	0.58	0.45	–	0.99
Viable pellet layer	q_s_, P/V, dO_2_	0.78	0.58	0.88	0.71
Mean specific productivity	q_s_	0.71	0.48	0.58	0.91

Ranges to differentiate good models according to MODDE: R2 (> 0.5), Q2 (> 0.1 for a significant model, > 0.5 for good model), Model validity (> 0.25), Reproducibility (> 0.5)

Morphological responses apart from pellet compactness are well described by MLR (see Table 2), these responses are dependent upon the factor power input. Issues in model fitting regarding pellet compactness can be explained by the low number of cultivations (only 3) featuring increased compactness due to a maximum power input over 2000 W/m^3^ in the uppermost region of the design space far from the normal operating range. These outliers lead to statistically significant model problems and low model validity. The impact of power input on morphology will be discussed in the detail in section: “Impact of power input on morphology”.

Viability and productivity are foremost dependent on the factor q_s_ which will be examined below in sections: “Impact of factors on viability” and “Interlink between productivity and specific substrate uptake”.

### Impact of power input on morphology

Morphological classification was performed as previously established by Ehgartner et al. [4]. This method enables classification according to hyphae, small clumps, large clumps and pellets. Summarising, gate setting is based on particle size in combination with SSC total to account for form of particles. In the following, pellets were analysed as most relevant morphological class as it encompasses 80–90% in relation to other classes.

**Fig. 5:**
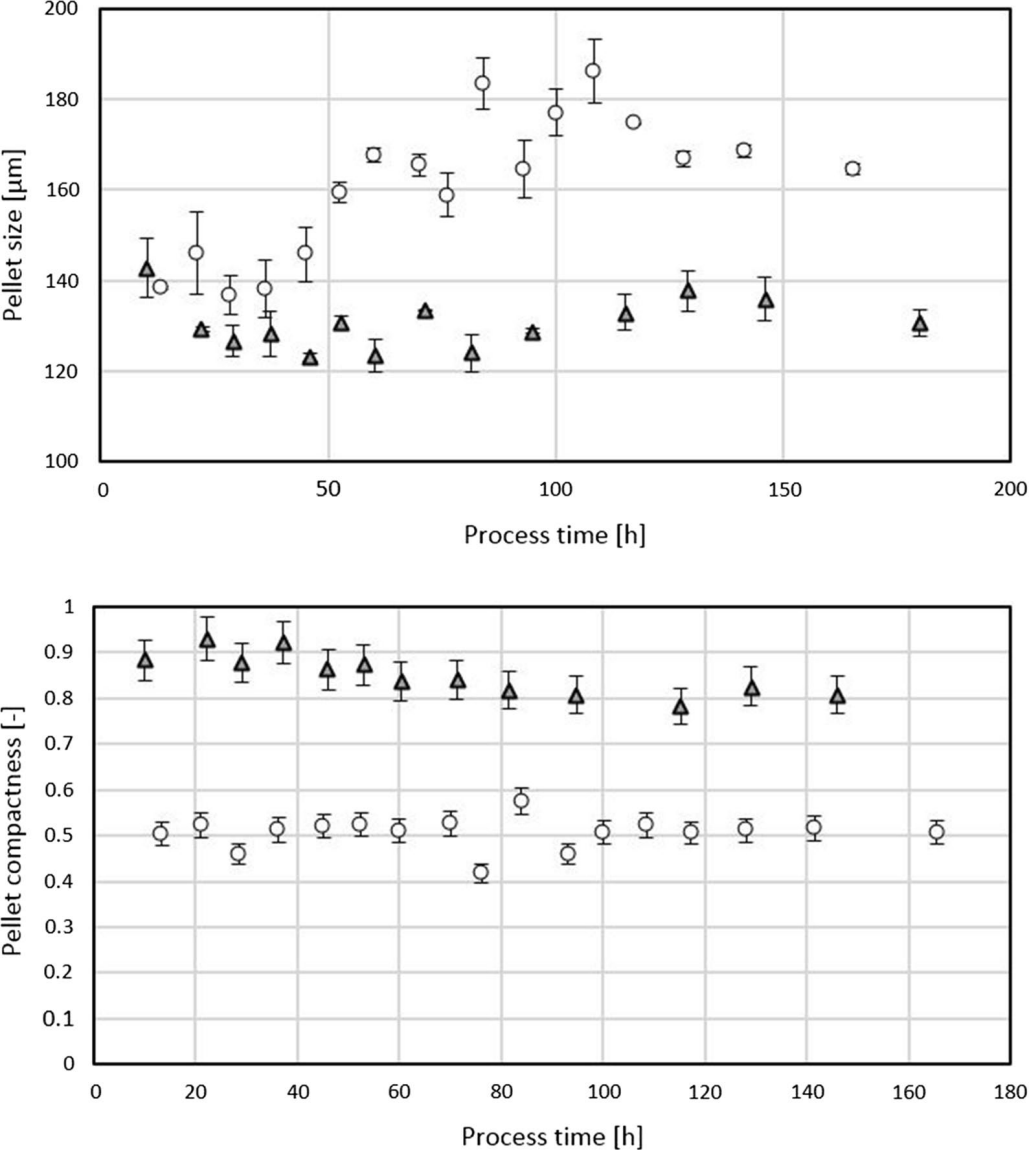
Morphological assessment of two bioreactor cultivations differing in power input. High power input at over 2000 W/m^3^ (triangles), low power input at 400 W/m^3^ (circles). Top: size of pellets, bottom: compactness of pellets across process time

Within Fig. 5 time resolved responses of two extreme power input points are presented. Both bioreactor cultivations are morphologically very diverse due to a considerably different power input controlled at either 400 W/m^3^ or 2000 W/m^3^. Average pellet size is increased by over 20 µm on average at lower power input. Compactness was calculated using SSC signals according to Eq. (6) as described by Veiter and Herwig [20]. Pellet compactness is greatly increased at power inputs exceeding 2000 W/m^3^.

**Fig. 6:**
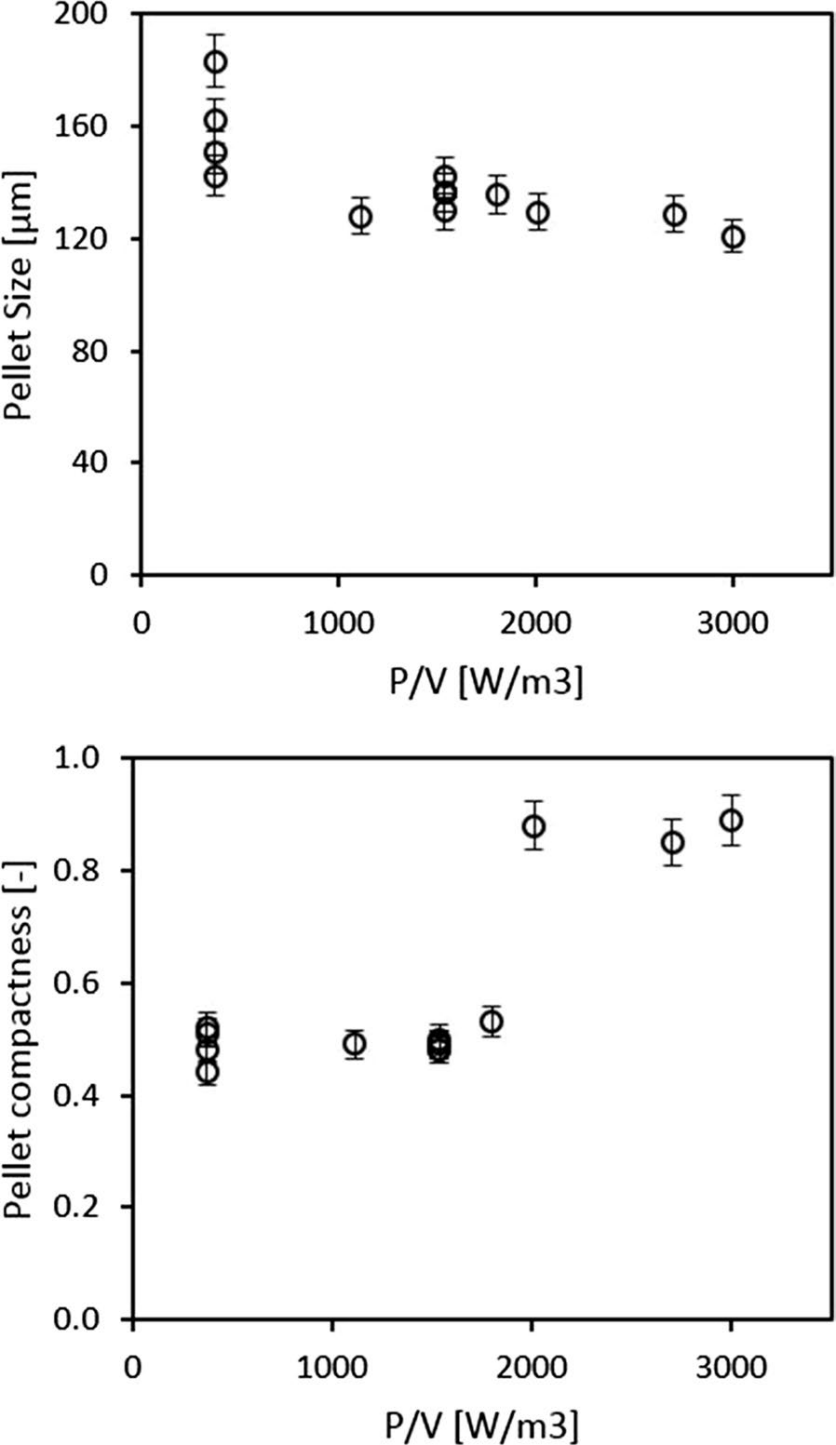
Dependence of morphological responses on power input, mean values from all bioreactor cultivations. Top: pellet size, bottom: pellet compactness

When looking at the entirety of bioreactor cultivations, Fig. 6 clearly demonstrates that all measured morphological responses are highly affected by the power input. Specifically, pellet fraction (in relation to all morphological classes) and pellet size are inversely proportional to power input while pellet compactness reacts proportionally. This is in accordance with literature [2], where agitation effects have been reported which can either break up the pellet (i), or shave-off the pellet’s hairy region (ii). Our results on pellet compactness are in accordance with the latter phenomenon. The impact of these morphological effects on viability will be further explained in section “Impact of factors on viability”.

### Impact of factors on viability

**Fig. 7:**
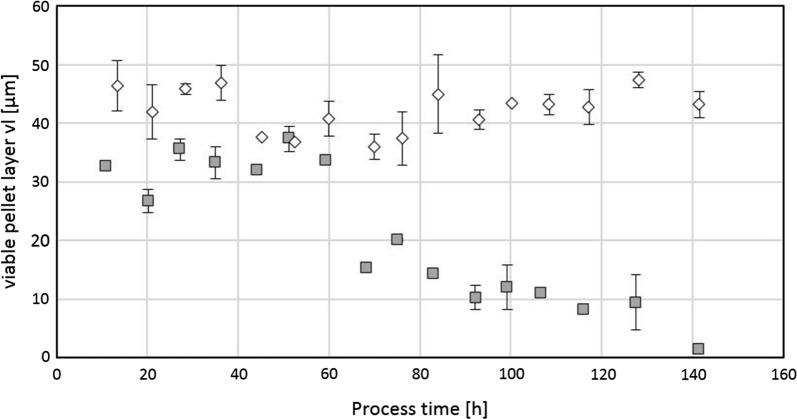
Viable layer of two bioreactor cultivations across process time. High q_s_ of 0.04 g_S_/g_X_/h and low dO_2_ content of 5% (grey rectangles), low q_s_ of 0.015 g_S_/g_X_/h and dO_2_ content of 40% (white diamonds)

As described in the introduction, we expected dependencies of the viable pellet layer on q_s_ and dO_2_. Trajectories of viable layer for two cultivations clearly display degradation of pellet biomass at high q_s_ and low dissolved oxygen content across process time (see Fig. 7).

These results clearly indicate that on the one hand the viable layer depends on dO_2_, however on the other hand oxygen and glucose consumption are very much interconnected: oxygen consumption is likely triggered by glucose consumption as described in “Introduction” section[1].

**Fig. 8:**
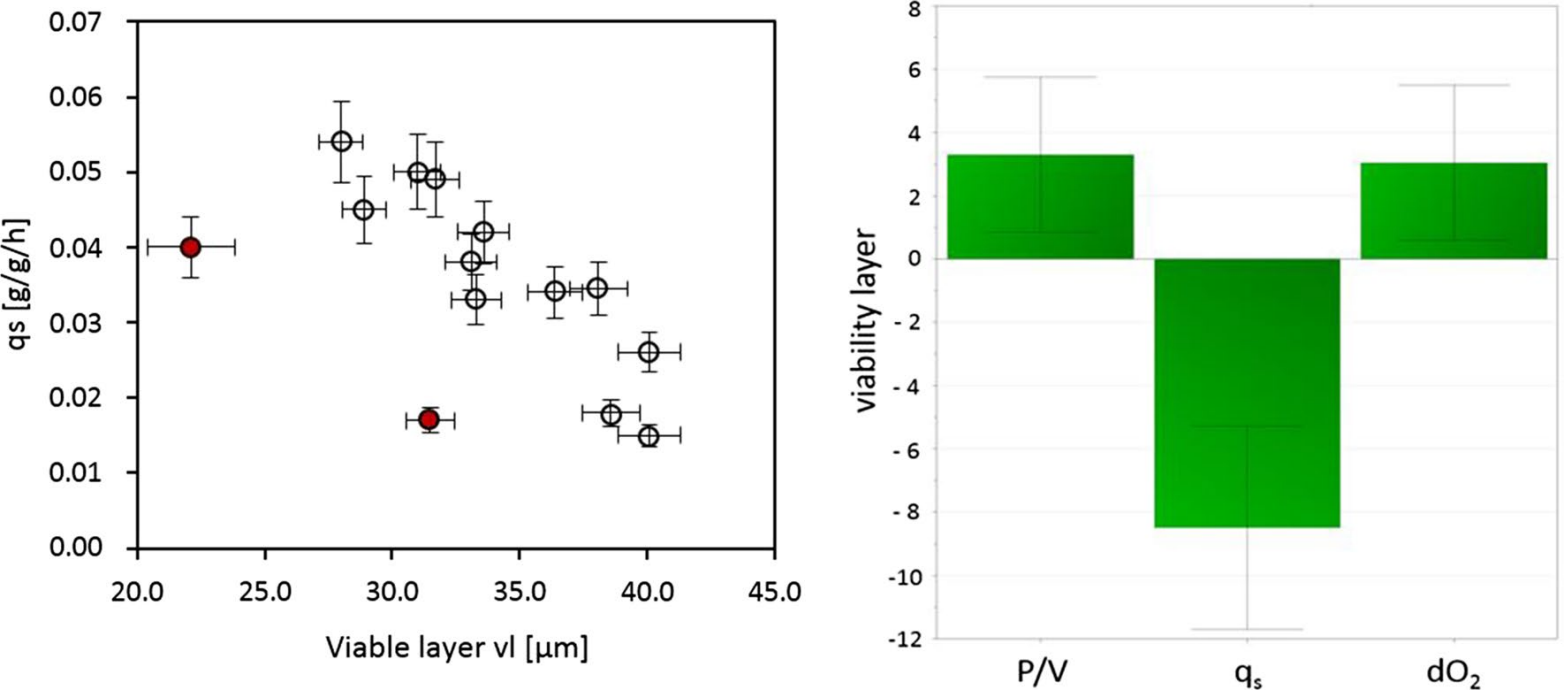
Dependence of viable layer on specific substrate uptake rate. Red triangles indicate bioreactor cultivations at low dissolved oxygen set points (left). Coefficient plot of the factors ‘P/V’, ‘q_s_’ and ‘dO_2_’ and their effects on the viable pellet layer (right). Interaction terms were omitted because they were not significant according to MODDE

Multiple linear regression further reveals the effects of q_s_ and dO_2_ on pellet viability. The coefficient plot (see Fig. 8) on the response viable layer reveals the negative impact of q_s_, which also represents the largest effect of all the factors. Consequently, the viable pellet layer is indirectly proportional to q_s_ as depicted in Fig. 8. As expected, we also observe a positive effect of dO_2_. Regarding the depicted advantageous effects of higher power inputs, we can remark that although dO_2_ was controlled via the gas mix, its control is still facilitated by a high power input with advantageous effects on mixing time and k_L_a [5].

To further estimate pellet viability and demonstrate the relation of viable layer to pellet size, a viability factor was calculated according to Eq. (7).

The effect of increased pellet compactness on diffusional limitations can be depicted in a correlation between compactness and diffusion factor adapted from Hille et al. [7]: in their contribution a so-called hyphal gradient in the pellet periphery was established which is comparable to the here-presented term pellet compactness. For the here presented data, the impact of increasing compactness on diffusion and furthermore viability is depicted in Additional file 1: Fig. S5. Results suggest that compactness levels exceeding 0.8 have negative effects on viability, however most bioreactor cultivations considered in this study feature lower compactness levels due to more moderate power inputs in the standard operating range.

Naturally, O_2_ diffusion is also highly dependent on dO_2_. Regarding the potential effect of a lack of O_2_ diffusion on pellet morphology, one can assume that low dissolved oxygen content leads to a collapse of O_2_ diffusion in the pellet’s inner region. Consequently, this would result in degradation of the pellet’s core and pellet breakage [4] as depicted in Fig. 3c.

These interlinks between substrate uptake, diffusional limitations related to morphology and dO_2_ not only affect viability but can be exploited favourably in order to increase productivity, which will be discussed in the following section.

### Interlink between productivity and specific substrate uptake

**Fig. 9:**
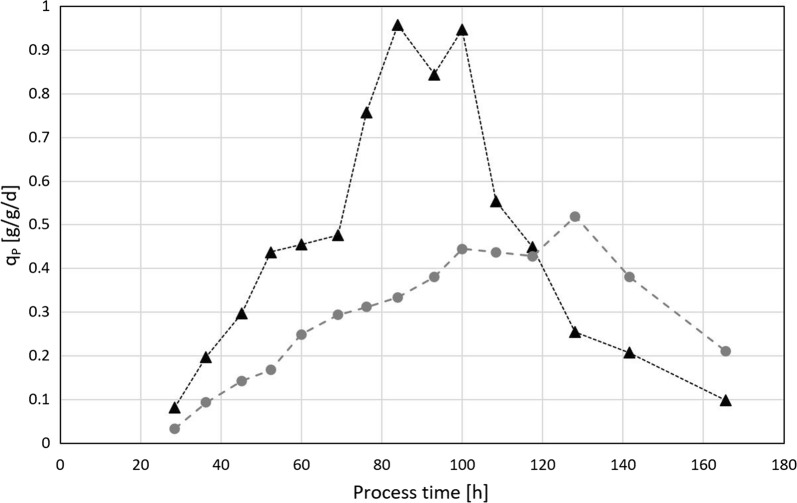
Trajectories of specific productivity over process time for cultivations at high specific substrate uptake rate q_s_ (black triangles) and low q_s_ (grey circles)

As demonstrated in Fig. 9, the trajectories of specific productivity (q_p_) reach a maximum and subsequently start to decline within 10–20 h of cultivation time. Each trajectory is dependent on the corresponding q_s_. Consequently, cultivations employing a high q_s_ reach their productivity maximum faster but also start to decline much earlier. This earlier decline phase is also reflected in a loss in viability as previously demonstrated in Fig. 8.

**Fig. 10:**
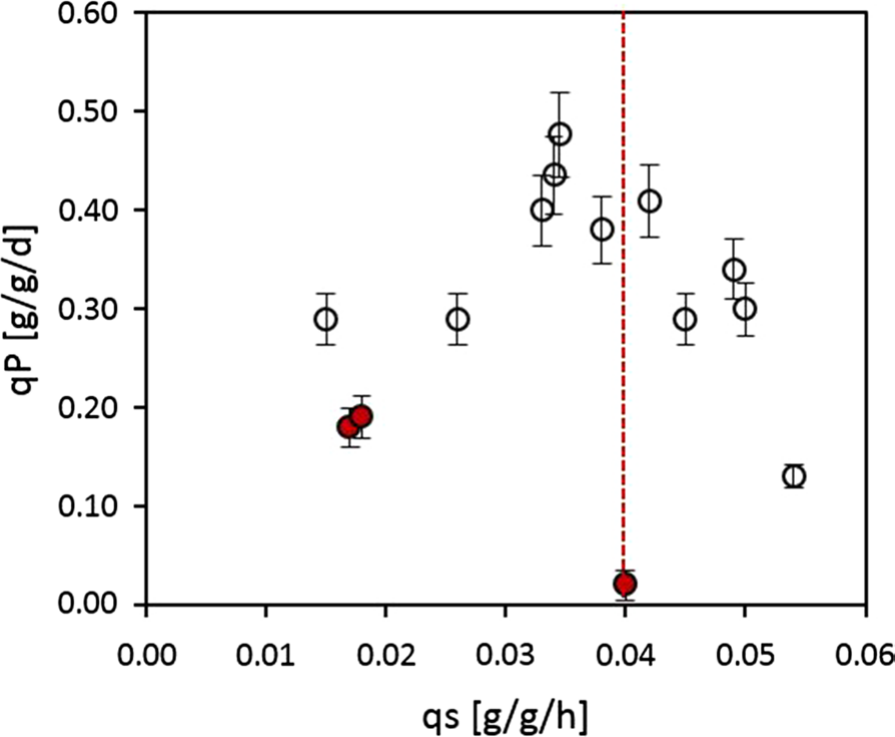
Dependence of specific productivity q_p_ on substrate uptake rate q_s_. Red triangles indicate bioreactor cultivations at low dissolved oxygen set points. Red dotted line indicates q_s_ threshold of q_P_ decline

When plotting the mean q_p_ against q_s_, a positive correlation is shown at low growth levels as depicted in Fig. 10. However, q_p_ declines at increasing q_s_ values. Literature suggests interlinks to the rate-limiting enzyme isopenicillin-N synthase, which is essential to penicillin production [3]. Our data indicates that the threshold for this decline in q_P_ is at a q_s_ of 0.04 g/g/h as already discussed in the MLR section. As a result, growth and production phases during cultivation should be based on q_s_. To ensure optimal productivity a q_s_ 0.03 g/g/h should never be exceeded. Fortunately, such a controlled qs also ensure high viability as previously discussed.

The response viable pellet layer is foremost dependent on q_s_. Similarly, q_s_ has also a considerable impact on q_p_. Both aspects can be used to determine an optimal operating range as depicted in the following section.

### Optimal process design space

**Fig. 11:**
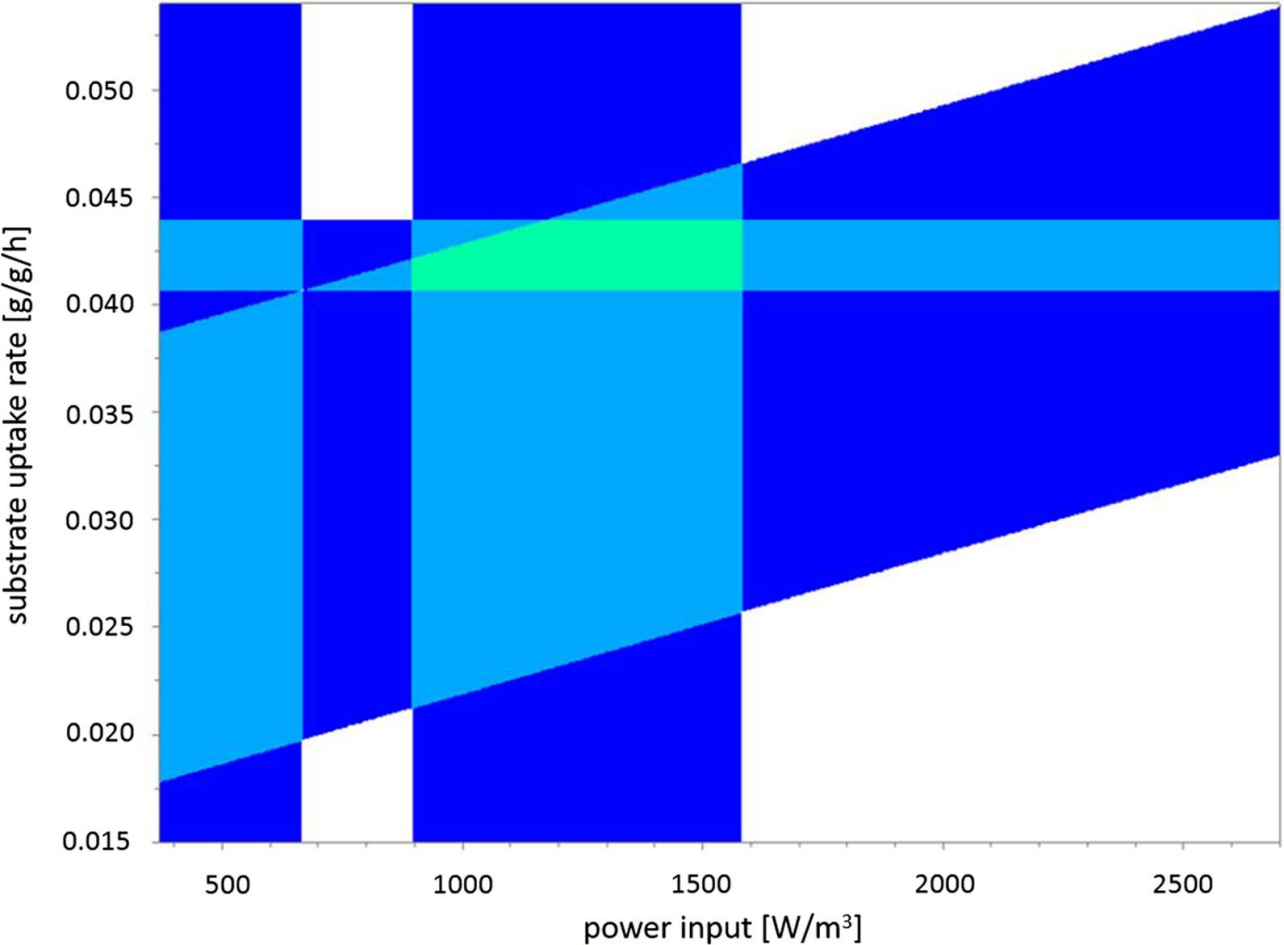
Sweet spot plot (green area) based on the following properties: viability factor: 0.6–0.75, viable layer: 31–40 µm, mean q_p_: 0.45–0.48, compactness: 0.5–0.6 at a pellet fraction: 75–95% of the whole biomass according to morphological classification. Dissolved oxygen content: 40%. Dark blue areas signify that two criteria are met; light blue areas signify that three criteria are met

Due the high number of interdependencies, optimisation efforts need to be performed with all DOE factors and corresponding responses in mind. A ‘sweet spot’ plot generated by MODDE displaying the optimum of q_s_ and P/V for a dO_2_ level of 40% is depicted in Fig. 11. Response ranges for this plot were set as: viable layer: 31–40 µm, mean q_p_: 0.45–0.48, compactness: 0.5–0.6. Note that the ‘sweet spot’ at these response ranges can only be achieved at the higher dO_2_ level of 40%.

The optimal design space was identified as follows: We were able to maintain a high number of pellets with favourable pellet compactness at a power input of 1500 W/m^3^. As demonstrated in section “Optimal process design space” (see Fig. 11), the compromise between viability and productivity is represented by a q_s_ of 0.040–0.045 g/g/h at the dO_2_ level of 40%.

**Fig. 12:**
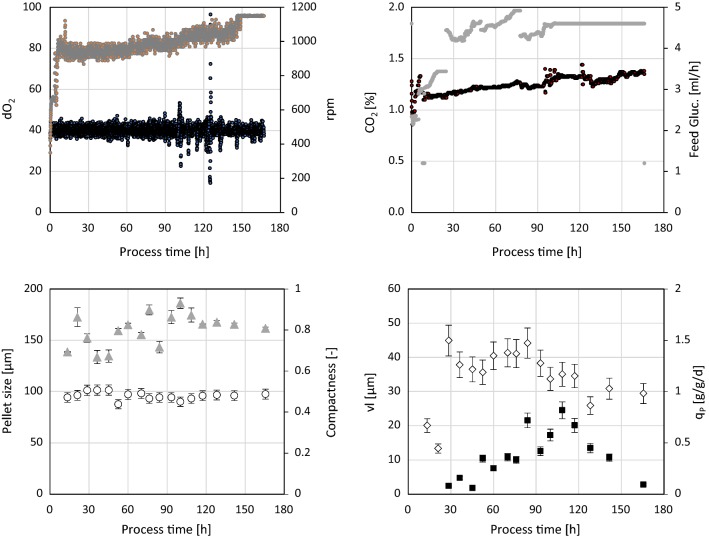
Cultivation MMH. Top: process data across process time: dO_2_ (black), agitation via rpm (grey), CO_2_ in off-gas (black), glucose feeding rate (grey). Bottom: responses across process time: pellet size (grey triangles), compactness (circles), viable layer (circles) and specific productivity (black rectangles)

Cultivation MMH meets the optimal operating range criteria, an overview on this cultivation is provided in Fig. 12. Mean pellet size was 136.5 ± 5.8 µm, mean compactness was 0.48 ± 0.02, mean viable layer was 33.1 ± 3.0 µm and mean specific productivity was 0.38 g/g/d. With a considerable standard deviation across process time of ± 0.17 q_p_ values of 0.7 g/g/d were well exceeded in this cultivation.

## Conclusions

From an industrial point of view, several aspects contribute to ensuring the maximum of process efficiency. The highest possible space–time-yield can be achieved via an optimised operating range of several factors: a feed regime dependent on q_s_ ensures a sound compromise between productivity and viability, at the same time favourable morphological conditions can be ensured through controlled power input. We found the optimal design space specifically tailored to our process which is presented in section “Optimal process design space” as: a q_s_ of 0.040–0.045 g/g/h at a power input of 1500 W/m^3^ and a dO_2_ level of 40%.

We were able to identify these advantageous fermentation parameters through a DoE approach in combination with novel morphological descriptors identified by flow cytometry analysis. For further process optimization, we envision a feeding profile with several q_s_ levels across process time starting with a high q_s_ of 0.05 g/g/h to quickly reach optimal q_p_-phases. To maintain a high q_p_ and viability q_s_ should be decreased below 0.02 g/g/h for the remainder of the cultivation process.

We envision the here-presented methodology to be suitable for any organism where process performance is highly dependent on morphology: for instance, we previously adapted the underlying flow cytometry-based method for physiological and morphological studies of glyco-engineered yeast [13].

## Supplementary information

**Additional file 1.** Additional figures and tables.

## Data Availability

The dataset(s) supporting the conclusions of this article are all included within the article. The datasets used and/or analyzed during the current study, if not shown in the text or additional files, are available from the corresponding author on reasonable request.

## References

[1] Bodizs L, Titica M, Faria N, Srinivasan B, Dochain D, Bonvin D (2007). Oxygen control for an industrial pilot-scale fed-batch filamentous fungal fermentation. J Process Control.

[2] Cui YQ, van der Lans RG, Luyben KC (1997). Effect of agitation intensities on fungal morphology of submerged fermentation. Biotechnol Bioeng.

[3] Douma RD, Verheijen PJ, de Laat WT, Heijnen JJ, van Gulik WM (2010). Dynamic gene expression regulation model for growth and penicillin production in *Penicillium chrysogenum*. Biotechnol Bioeng.

[4] Ehgartner D, Herwig C, Fricke J (2017). Morphological analysis of the filamentous fungus *Penicillium chrysogenum* using flow cytometry-the fast alternative to microscopic image analysis. Appl Microbiol Biotechnol.

[5] Garcia-Ochoa F, Gomez E (2009). Bioreactor scale-up and oxygen transfer rate in microbial processes: an overview. Biotechnol Adv.

[6] Hille A, Neu TR, Hempel DC, Horn H (2005). Oxygen profiles and biomass distribution in biopellets of *Aspergillus niger*. Biotechnol Bioeng.

[7] Hille A, Neu TR, Hempel DC, Horn H (2009). Effective diffusivities and mass fluxes in fungal biopellets. Biotechnol Bioeng.

[8] Kager J, Herwig C, Stelzer IV (2018). State estimation for a penicillin fed-batch process combining particle filtering methods with online and time delayed offline measurements. Chem Eng Sci.

[9] Nielsen J, Johansen CL, Jacobsen M, Krabben P, Villadsen J (1995). Pellet formation and fragmentation in submerged cultures of *Penicillium chrysogenum* and its relation to penicillin production. Biotechnol Prog.

[10] Paul GC, Syddall MT, Kent CA, Thomas CR (1998). A structured model for penicillin production on mixed substrates. Biochem Eng J.

[11] Paul GC, Thomas CR (1996). A structured model for hyphal differentiation and penicillin production using *Penicillium chrysogenum*. Biotechnol Bioeng.

[12] Paul GC, Thomas CR (1998). Characterisation of mycelial morphology using image analysis. Adv Biochem Eng Biotechnol.

[13] Pekarsky A, Veiter L, Rajamanickam V, Herwig C, Grünwald-Gruber C, Altmann F, Spadiut O (2018). Production of a recombinant peroxidase in different glyco-engineered *Pichia pastoris* strains: a morphological and physiological comparison. Microb Cell Fact.

[14] Pirt SJ, Righelato RC (1967). Effect of growth rate on the synthesis of Penicillin by *Penicillium chrysogenum* in batch and chemostat cultures. Appl Microbiol.

[15] Posch AE, Herwig C (2014). Physiological description of multivariate interdependencies between process parameters, morphology and physiology during fed-batch penicillin production. Biotechnol Prog.

[16] Rutherford K, Mahmoudi SMS, Lee KC, Yianneskis M (1996). The influence of Rushton impeller blade and disk thickness on the mixing characteristics of stirred vessels. Chem Eng Res Des.

[17] Stelzer IV, Kager J, Hervig C (2017). Comparison of particle filter and extended kalman filter algorithms for monitoring of bioprocesses. 27th European symposium on computer aided process engineering, Pt B 40b: 1483–1488.

[18] Tayeb YJ, Lim HC (1986). Optimal glucose feed rates for fed-batch penicillin fermentation—an efficient algorithm and computational results. Ann N Y Acad Sci.

[19] Vansuijdam JC, Metz B (1981). Fungal pellet breakup as a function of shear in a fermenter. J Ferment Technol.

[20] Veiter L, Herwig C (2019). The filamentous fungus *Penicillium chrysogenum* analysed via flow cytometry-a fast and statistically sound insight into morphology and viability. Appl Microbiol Biotechnol.

[21] Veiter L, Rajamanickam V, Herwig C (2018). The filamentous fungal pellet-relationship between morphology and productivity. Appl Microbiol Biotechnol.

[22] Walisko R, Moench-Tegeder J, Blotenberg J, Wucherpfennig T, Krull R (2015). The taming of the shrew-controlling the morphology of filamentous eukaryotic and prokaryotic microorganisms. Adv Biochem Eng Biotechnol.

[23] Zhang J, Zhang J (2016). The filamentous fungal pellet and forces driving its formation. Crit Rev Biotechnol.

