# Supplementary material for: Optimal process design space to ensure maximum viability and productivity in Penicillium chrysogenum pellets during fed-batch cultivations through morphological and physiological control
Source: Microb Cell Fact. 2020 Feb 13;19:33. doi: 10.1186/s12934-020-1288-5 (PMC7020361; doi:10.1186/s12934-020-1288-5)
Supplement: Supplementary file 1 — Additional file 1. Additional figures and tables. [file 12934_2020_1288_MOESM1_ESM.docx]

**Additional file**

**Optimal process design space to ensure maximum viability and productivity in *Penicillium chrysogenum* pellets during fed-batch cultivations through morphological and physiological control**

Lukas Veiter^1,2^, Julian Kager^1^ and Christoph Herwig*^,1,2^

*to whom the correspondence should be addressed to

^1^ CD Laboratory on Mechanistic and Physiological Methods for Improved Bioprocesses, Vienna University of Technology, Gumpendorferstrasse 1a/166, 1060 Vienna, Austria

^2^ Research Area Biochemical Engineering, Institute of Chemical, Environmental and Bioscience Engineering, Vienna University of Technology, Gumpendorferstrasse 1a/166, 1060 Vienna, Austria

*Corresponding author:

christoph.herwig@tuwien.ac.at; Tel (Office): +43 1 58801 166400

Gumpendorferstrasse 1a / 166-4

1060 Wien, Austria

**Table S1.** Single-factor ANOVA analysis (α = 0.05) of responses

| Response | F - value | p – value | Critical F - value |
| --- | --- | --- | --- |
| Pellet size | 71.0 | 9.9 * 10^-61g^ | 1.97 |
| Pellet compactness C | 529.3 | 5.5 * 10^-630^ | 1.97 |
| Viable layer vl | 20.3 | 9.4 * 10^-230^ | 1.98 |
| Specific productivity q_p_ | 8.9 | 1.8 * 10^-900^ | 2.02 |


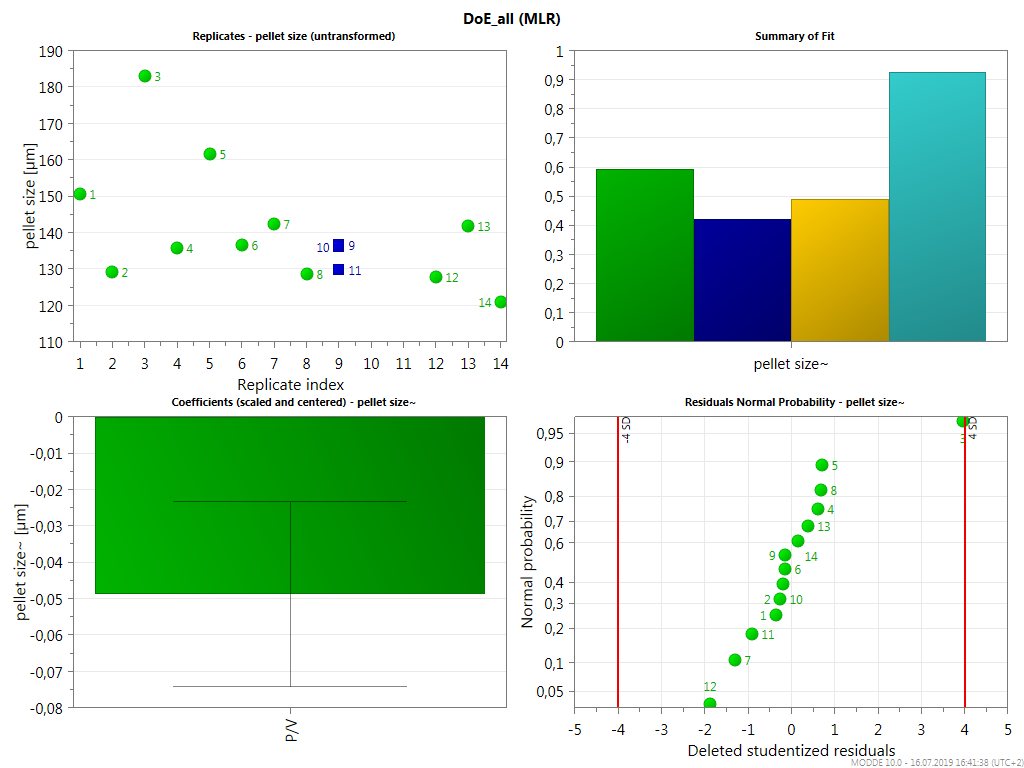


Figure S1. Overview for response pellet size: replicates, summary of fit, coefficients, residuals normal probability.


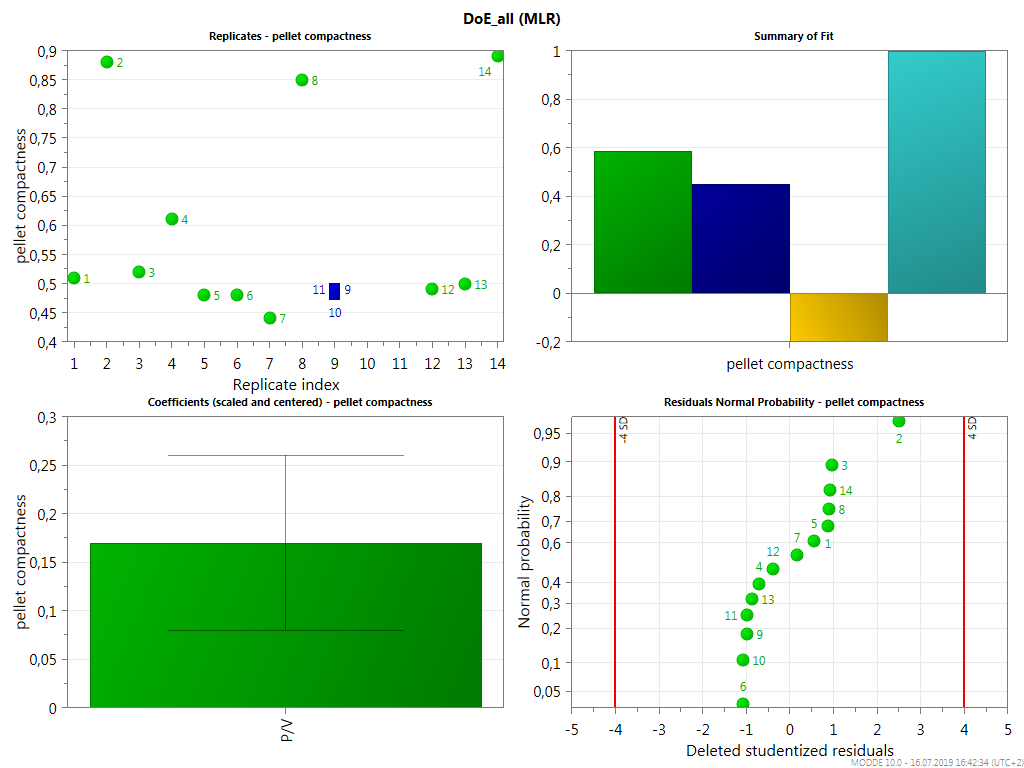


Figure S2. Overview for response pellet compactness: replicates, summary of fit, coefficients, residuals normal probability.


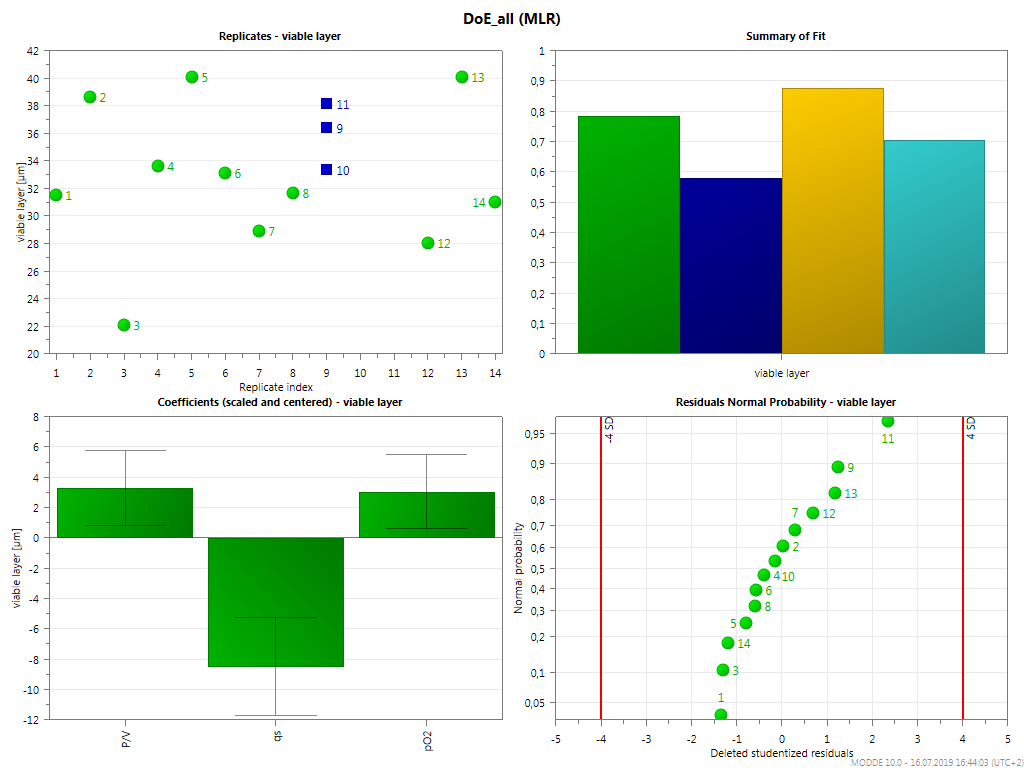


Figure S3. Overview for response viable layer: replicates, summary of fit, coefficients, residuals normal probability.


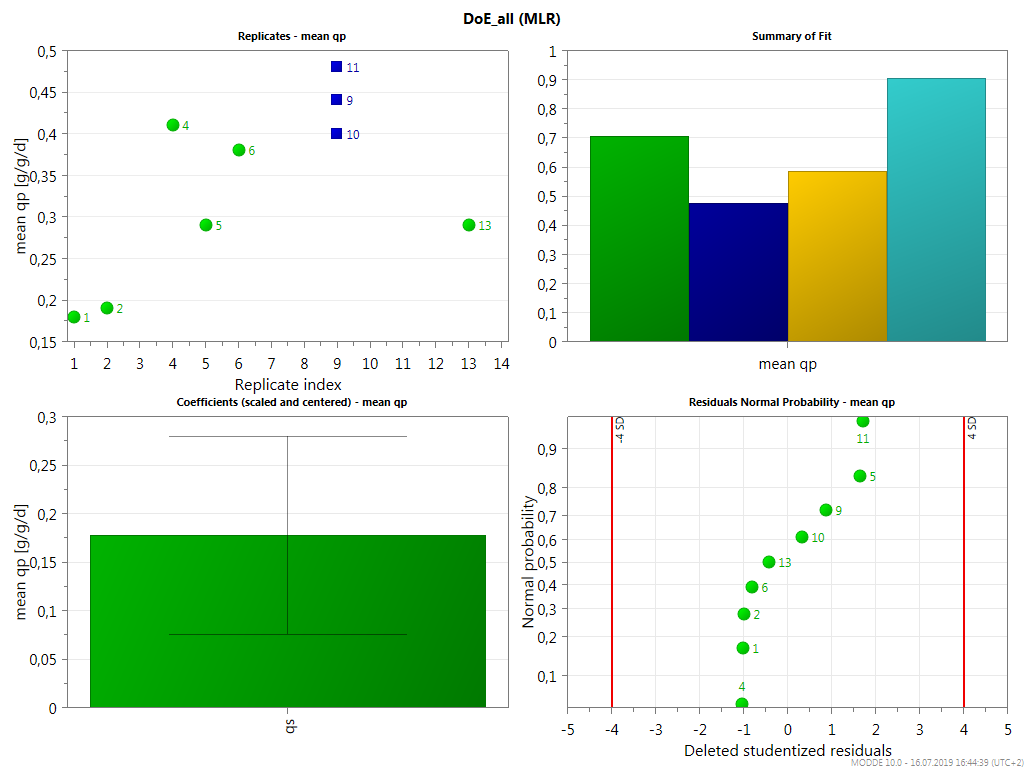


Figure S4. Overview for response mean q_P_: replicates, summary of fit, coefficients, residuals normal probability.

**Figure S5.** Impact of compactness on diffusion factor (top) and viability factor (bottom). Red circles indicate bioreactor cultivations at low dO_2_ set points.
